# Association of ApoE Genetic Polymorphism and Type 2 Diabetes with Cognition in Non-Demented Aging Chinese Adults: A Community Based Cross-Sectional Study

**DOI:** 10.14336/AD.2017.0715

**Published:** 2018-06-01

**Authors:** Jie Zhen, Tong Lin, Xiaochen Huang, Huiqiang Zhang, Shengqi Dong, Yifan Wu, Linlin Song, Rong Xiao, Linhong Yuan

**Affiliations:** School of Public Health, Capital Medical University, Beijing 100069, China; School of Public Health, Capital Medical University, Beijing 100069, China; School of Public Health, Capital Medical University, Beijing 100069, China; School of Public Health, Capital Medical University, Beijing 100069, China; School of Public Health, Capital Medical University, Beijing 100069, China; School of Public Health, Capital Medical University, Beijing 100069, China; School of Public Health, Capital Medical University, Beijing 100069, China; School of Public Health, Capital Medical University, Beijing 100069, China; School of Public Health, Capital Medical University, Beijing 100069, China

**Keywords:** apolipoprotein E, polymorphism, type 2 diabetes mellitus, cognitive function, geriatrics

## Abstract

Apolipoprotein E (ApoE) gene polymorphism has been implicated in predisposition to diabetes and dementia in old population, but the results from the different studies were inconclusive. A cross-sectional study was carried out to explore the relationship among ApoE gene polymorphism, diabetes and cognition in non-demented aging Chinese adults. A total number of 1000 community dwellers aged 55 years and above were randomly recruited. Demographic information of the participants was collected using well designed self-administered questionnaires. The Montreal Cognitive Assessment (MoCA) test was employed to evaluate the cognitive status of the participants. Semi-quantitative food frequency questionnaire was used to obtain the dietary intake information. Fasting venous blood samples were taken for ApoE genotyping and serum lipid measurements. 238 participants were type 2 diabetes mellitus (T2DM) patients and 145 participants were *ApoE4* carriers. *ApoE 4*-T2DM subjects had higher serum triglyceride (TG) concentration than *E2* and *E3* carriers (*P* < 0.05). T2DM subjects carrying *ApoE4* had lower cognition than subjects with *E2* or *E3* carriers (*P* < 0.05). Comparing to non-type 2 diabetic mild cognitive impaired (nT2DM-MCI) subjects, the type 2 diabetic mild cognitive impaired (T2DM-MCI) subjects have higher serum glucose (Glu) level and lower high-density lipoprotein (HDL-C) level (*P* < 0.05). The T2DM-MCI subjects carrying *ApoE4* have lower cognition than *E2* and *E3* carriers (*P* <0.05); and the interaction of *ApoE* genotype with T2DM was detected (*P* < 0.05). Our results indicated the association among ApoE gene polymorphism, T2DM and cognitive performance in non-demented aging population. The carrying of *ApoE4* predisposed the T2DM subjects and the T2DM-MCI subjects to have poor cognitive performance. Additional experimental studies are required to explore the mechanism that *ApoE* genotype modifies the risk for cognitive impairment in aging subjects with T2DM.

Type 2 diabetes mellitus (T2DM) is one of the most common chronic metabolic diseases throughout the world. It was estimated that more than 300 million people worldwide were affected by T2DM [1]. In 2010, the prevalence of T2DM was reported to be 11.6% in Chinese population [2]. Furthermore, the high incidence of Alzheimer’s disease (AD) was observed in T2DM patients [3-5]. Epidemiology studies indicated that patients with T2DM had a 1.5-2.0-fold increased risk for developing dementia than those without T2DM [6,7]. T2DM, cognition decline and Alzheimer’s disease (AD) are all age-associated progressive disability disorders with high prevalence in the elderly. The older population is growing dramatically in China. Therefore, in order to decrease the incidence of dementia in Chinese population, the prevention and control of T2DM are becoming much more urgent and imperative.

The underlying mechanisms through which that T2DM influencing cognitive functions in aging population are unclear [8]. Many factors such as gender, ethnicity and body weight were suggested to be associated with cognition changes in T2DM patients [9-11]. Additionally, as a typical diabetic phenotype, metabolic dyslipidemia was suggested to have strong correlation with cognition decline in aging subjects with diabetes [12]. Apolipoprotein E (*ApoE*) is a candidate gene for the development of T2DM due to its critical role in the lipid metabolism. Correlations between ApoE gene polymorphism and the pathogenesis of T2DM have been indicated in previous studies [13,14]. Besides, ApoE was proved to participate in the lipid metabolism and transport of cholesterol in brain [15]. Adverse associations of the *ApoE ε4* allele have also been reported for the development of AD [16]. Earlier age of onset and a more rapid progression of the disease were usually founded in AD patients with *ApoE ε4* allele [17]. Data from experimental animal studies also indicated that the ApoE 4 isoform involved in the formation of β-amyloid (Aβ) plaques and neurofibrillary tangles (the two main neuropathological hallmarks of AD) in the pathogenesis of AD [18]. Human beings-based studies indicated that the ApoE4 carrier exhibit reduced clearance of Aβ and insufficient neuronal damage repair compared to the E2 and E3 carriers [19]. Recently, the increased risk for dementia in T2DM patients was also shown to be associated with *ApoE* genotypes [20]. T2DM patients carrying one or two *ApoE ε4* allele had a significantly higher risk of dementia than those who were negative for T2DM or *ApoE ε4* allele [21].

Increased convincing evidences indicated the risk of cognitive impairment and AD in T2DM patients. Yet, the relationships between cognitive function and T2DM in non-demented elderly people remain insufficiently explored. ApoE gene polymorphisms have also been implicated in predisposition to diabetes and dementia, but the results from the different studies were inconclusive. Especially, few studies explore the association of ApoE genetic variants, T2DM with cognition in aging Chinese adults. Thus, in the present work, a community based cross-sectional study was carried out aiming to explore how ApoE gene polymorphism and T2DM associates with cognitive changes, as well as whether ApoE genetic polymorphism modulates the association between T2DM and cognitive function in non-demented community aging Chinese adults.

## MATERIALS AND METHODS

### Participants

A community-based cross-sectional study was carried out from April of 2012 to April of 2013. The design protocol was approved by the Human Ethics Committee of the Capital Medical University (No. 2012SY23). The procedures followed the ethical standards of the Helsinki Declaration of 1975. A total number of 1000 community dwellers aged 55 years and above were randomly recruited by advertisements and direct phone dialing by the nurses from Nanyuan and Wulituo Community service centers, Beijing, China. Subjects with conditions known to affect cognitive function (e.g., alcohol abuse, history of cerebral apoplexy or cerebral infarction); as well as subjects with AD, Parkinson’s disease (PD), long-term frequency intake of antidepressants and medication acting on central nervous system were also excluded from the present study. Diabetes was ascertained based on the self-reported medical history by the participants. Written informed consent was obtained from all enrolled participants.

### Socio-demographic Variables and Anthropometric Measurements

Anthropometric measures, including height and weight, were documented by the nurses from the community medical service center. BMI was calculated as weight (kg)/height (m^2^). Educational level was assessed as the highest level attained and classified into six categories (illiterate, primary school, junior high school, high school, junior college, undergraduate and above). Information on demographic characteristics (gender, age), lifestyle factors [e.g., smoking (yes or no), alcohol drinking (yes or no), physical activity (never, 1-3 times/wk, 4-5 times/wk, everyday)], living status (living alone, yes or no), reading habit (yes or no) and housework doing (yes or no) was collected by using a well structured self-administered questionnaire.

### Cognitive Test

Global cognitive function was assessed with the Montreal Cognitive Assessment (MoCA) by medical doctors from the community health service center. MoCA test consists of seven cognitive domains including visual-spatial and executive ability, naming, attention, abstraction, language, delayed memory recall and orientation functions. The MoCA appears to have utility as a cognitive screening tool with high sensitivity and specificity for early detection of MCI [22,23]. According to previous study conducted in Chinese older population [24], the cut-off points used for mild cognitive impairment (MCI) diagnosis were as follow: 13/14 for individuals with no formal education, 19/20 for individuals with 1 to 6 years of education, and 24/25 for individuals with 7 or more years of education. The cut-offs above were proved sensitive and efficient in the diagnosis of MCI in older Chinese population.

### Dietary Assessment

Participants were visited at a community health service center by specifically trained nutritionists and registered nurses. A validated semi-quantitative food frequency questionnaire (FFQ) was used to assess the habitual consumption of 10 food groups (fruit and vegetable, whole grain, legume, red meat, poultry, fish, eggs, nuts, cooking oil, milk, comprising 35 items in total). This questionnaire was adopted from a questionnaire used for the Dietary Investigation of Chinese Residents, which was organized by the Chinese Nutrition Society (CNS) [25]. The food intake survey documented the information, including the consumption frequencies (daily and weekly) and the amount of foods consumed.

### DNA Isolation and Genotyping

Peripheral blood samples (6 ml intravenously) were collected in vacuum tubes and stored at -80?. DNA was extracted from frozen peripheral blood using the Wizare genomic DNA purification kit (Promega, Madison, WI, USA). *ApoE* genotypes were determined by Polymerase Chain Reaction (PCR) amplification and Restricted Fragment Length Polymorphism (RFLP) analysis according to the method described by Hixson [24]. The specific primers used for *ApoE* genotyping are: forward, 5′-GGC ACG GCT GTCCAA GGA-3′; reverse, 5′-GCC CCG GCC TGG TAC ACT GCC-3′. In addition, 20% of DNA samples were genotyped again by different operators for the purpose of quality control of the genotyping.

### Serum Parameter Measurement

Blood samples were drawn after 12 hour (h) fasting. Then, centrifuged at 1500 g for 15 minutes at 4?, serum was separated within 2 h, and all samples were stored at 40? until further laboratory tests. An ILAB600 clinical chemistry analyzer (Instrumentation Laboratory, Lexington, WI, USA) was used to determine serum total cholesterol (TC) and triglyceride (TG). High density lipoprotein cholesterol (HDL-C) was measured by using a commercially available assay from Instrumentation Laboratory (Lexington, WI, USA). Low density lipoprotein cholesterol (LDL-C) was calculated by using the Friedewald formula [27]. All samples for each participant were analyzed within a single batch, and the inter-assay coefficients of variation (CV) were less than 5%.

### Statistical Analyses

Data was analyzed with the software SPSS 19.0 (Chicago, IL, USA). Continuous variables were presented as mean (95% confidence interval, CI) or means ± standard deviation (SD). Gender, smoking, alcohol drinking, physical activity, living status, reading habit and housework doing were presented as category variables. Participants were classified according to categories of *ApoE* alleles. General linear model (GLM) was used to compare the means of the detected parameters between the groups. The following putative confounding factors were included in the analyses: age, gender, body mass index (BMI), education level (schooling completed), physical activity (never, 1-3 times/week, 4-5 times/week, everyday), smoking (yes or no), alcohol drinking (yes or no), living status (living alone, yes or no), reading habit (yes or no) and housework doing (yes or no). P < 0.05 was considered to be statistically significant.

## RESULTS

### Demographics of the Participants

Initially, a total of 1000 older Chinese adults participated in the present study. 48 subjects were excluded due to uncompleted questionnaires, unsuccessful biological specimen sampling or unsuccessful genotyping. A total of 952 participants were in the final sample analysis. For ApoE genotypes, subjects with the *E2/E2* and *E2/E3* genotypes were grouped as *E2* carrier; subjects with *E3/E3* were classified as *E3* homozygote; and subjects with *E3/E4* or *E4/E4* were grouped as *E4* carrier. 25.0% of the subjects were classified as having type 2 diabetes mellitus (T2DM); and 15.23% of the participants were *ApoE4* carrier. There was no statistical significance of demographic factors including age, gender, BMI, education level and lifestyle between T2DM and non-T2DM (nT2DM) subjects (*P* > 0.05). Compared with subjects without T2DM, those with T2DM have higher serum Glu, TG levels and lower HDL-C level (*P* < 0.05). Of all T2DM subjects, 13.87% of the subjects were *E4* carriers. No statistical significance of *ApoE* genotype frequencies and cognition was detected between nT2DM and T2DM subjects (*P* > 0.05) (Table 1 & 2).

### Serum parameters and cognition by T2DM in MCI subjects

Totally, 211 subjects were diagnosed as MCI according to the cut-off of MoCA score. And we categorized the MCI subjects into nT2DM-MCI group and T2DM-MCI group. As shown in Table 3, comparing to nT2DM-MCI subjects, the T2DM-MCI subjects have higher serum Glu level and lower HDL-C level (*P* < 0.05). No significant difference of *ApoE* allele frequencies and cognition was detected between nT2DM and T2DM-MCI subjects (*P* > 0.05).

**Table 1:** Demographics of the participants.

Demographic character	T2DM		Total *(n = 952)*	*P* value
	No *(n = 714)*	Yes *(n = 238)*		
Age, mean ± SD	62.8 ± 5.8	63.2 ± 5.7	62.9 ± 5.8	0.31
Gender, n (%)				0.17
*male*	219 (30.7)	85 (35.7)	304 (31.9)	
*Female*	495 (69.3)	153 (64.3)	648 (68.1)	
BMI (kg/m^2^), mean ± SD	25.5 ± 7.6	25.46 ± 3.4	25.5 ± 6.8	0.87
Education, n (%)				0.45
*Illiterate*	23 (3.2)	12 (5.0)	35 (3.7)	
*Primary school*	107 (15.0)	42 (17.6)	149 (15.7)	
*Junior high school*	328 (45.9)	114 (47.9)	442 (46.4)	
*High school*	190 (26.6)	53 (22.3)	243 (25.5)	
*Junior college*	42 (5.9)	11 (4.6)	53 (5.6)	
*Undergraduate and above*	24 (3.4)	6 (2.5)	30 (3.2)	
Life style				
Living alone, n (%)				0.87
*Yes*	44 (6.2)	13 (5.5)	57 (6.0)	
*No*	670 (93.8)	225 (94.5)	895 (94.0)	
Smoking, n (%)				0.24
*Yes*	110 (15.4)	42 (14.6)	152 (16.0)	
*No*	604 (84.6)	196 (82.4)	800 (84.0)	
Alcohol drinking, n (%)				0.67
*Yes*	199 (27.9)	63 (26.5)	262 (27.5)	
*No*	501 (72.1)	175 (73.5)	690 (72.5)	
Physical activity, n (%)				0.14
*Never*	73 (10.2)	25 (10.5)	98 (10.3)	
*1-3 times/week*	98 (13.7)	23 (9.7)	121 (12.7)	
*4-5 times/week*	91 (12.8)	22 (9.2)	113 (11.9)	
*everyday*	451 (63.2)	168 (70.6)	619 (65.1)	
Reading habit, n (%)				0.15
*Yes*	342 (47.9)	100 (42.0)	442 (46.4)	
*No*	372 (52.1)	138 (58.0)	510 (53.6)	
Housework doing, n (%)				0.15
*Yes*	676 (94.7)	220 (92.4)	896 (94.3)	
*No*	38 (5.3)	18 (7.6)	56 (5.9)	

BMI, body mass index; Glu, glucose; HDL-C, high density lipoprotein cholesterol; LDL-C, low density lipoprotein cholesterol; TC, total cholesterol; TG, triglyceride; ApoE, apolipoprotein E; T2DM: type 2 diabetes mellitus. Demographic characteristics including age and BMI were compared by using t- tests. Demographic characteristics including gender, education, lifestyle and *Apo*E allele frequencies were compared by using Chi-square test.

### Dietary intake of MCI subjects with or without T2DM

Dietary intake of nT2DM-MCI subjects and T2DM-MCI subjects were compared in Table 4. We only detected the difference of daily legume intake between the groups (*P* < 0.05). For other food items, no statistical significance was observed between the groups (*P* > 0.05).[Table T2-ad-9-3-346]

**Table 2 T2-ad-9-3-346:** Serum parameter, ApoE genotype and cognition of the participants.

Parameter, genotype and cognition	T2DM		Total *(n = 952)*	*P* value
	No *(n = 714)*	Yes *(n = 238)*		
Serum parameters (mmol/L), mean (95% *CI*)			
*Glu*	5.4 (5.3, 5.5)	7.2 (7.0, 7.4)	6.3 (6.2, 6.4)	0.00
*TC*	5.1 (5.0, 5.2)	5.0 (4.8, 5.1)	5.0 (5.0, 5.1)	0.12
*TG*	1.8 (1.7, 1.9)	2.0 (1.9, 2.2)	1.9 (1.8, 2.0)	0.00
*LDL-C*	3.2 (3.1, 3.2)	3.2 (3.1, 3.3)	3.2 (3.1, 3.2)	0.67
*HDL-C*	1.4 (1.4, 1.4)	1.3 (1.3, 1.3)	1.3 (1.3, 1.4)	0.00
*ApoE* genotype, n (%)				0.68
*E2*	105 (14.7)	29 (12.2)	134 (14.1)	
*E3*	497 (69.6)	176 (74.0)	673 (70.7)	
*E4*	112 (15.7)	33 (13.9)	145 (15.2)	
Cognition				
*Visual & executive*	3.91 (3.8, 4.0)	3.8 (3.7, 4.0)	3.9 (3.8, 4.0)	0.37
*Naming*	2.9 (2.9, 2.9)	2.9 (2.9, 3.0)	2.9 (2.8, 2.9)	0.91
*Attention*	5.3 (5.2, 5.4)	5.4 (5.3, 5.6)	5.4 (5.3, 5.5)	0.09
*Language*	2.2 (2.1, 2.3)	2.2 (2.1, 2.4)	2.2 (2.2, 2.3)	0.58
*Abstraction*	1.6 (1.5, 1.7)	1.6 (1.5, 1.7)	1.6 (1.6, 1.7)	0.69
*Memory and delayed recall*	3.0 (2.9, 3.1)	3.1 (2.9, 3.3)	3.0 (2.9, 3.1)	0.30
*Orientation*	5.8 (5.7, 5.8)	5.8 (5.7, 5.9)	5.8 (5.7, 5.8)	0.63
*MoCA score*	24.9 (24.5, 25.2)	25.2 (24.6, 25.8)	25.1 (24.7, 25.4)	0.29

Data were expressed as mean (95% *CI*). Glu, glucose; HDL-C, high density lipoprotein cholesterol; LDL-C, low density lipoprotein cholesterol; TC, total cholesterol; TG, triglyceride; ApoE, apolipoprotein E; T2DM: type 2 diabetes mellitus; MCI, mild cognitive impairment. *ApoE* genotype frequencies were compared by using Chi-square test. General Line Model (GLM) was used for data serum parameters and cognition comparison. During the comparison of serum parameters and cognition between groups, factors including age, BMI, education, smoking, alcohol drinking, exercise, reading habits, housework doing were adjusted. *P* < 0.05 was considered as significance.

### Serum parameters and cognition by ApoE genotype in T2DM subjects

As shown in Table 5, for serum TG concentration, the T2DM subjects carrying *ApoE4* have higher serum TG concentration than *ApoE2* carrier, and even higher than those with *ApoE3* homozygote (*P* < 0.05). Cognition of the T2DM subjects was significantly different by *ApoE* genotypes. The *ApoE2*-T2DM subjects had higher abstraction ability than subjects with *ApoE3* homozygote, and much higher than *ApoE4* carrier (*P* < 0.05). For global cognition, the *ApoE4*-T2DM subjects had the lowest total MoCA score than *ApoE2* carrier and those with *ApoE3* homozygote (*P* < 0.05).

### Combine effect of ApoE genotype and T2DM on serum parameters and cognition in aging Chinese adults

After grouping the participants according to *ApoE* genotype and whether they were T2DM patients, we did not detect the synergistic effect of *ApoE* genotype and T2DM on serum parameter status and cognition (*P* > 0.05) (Table 6).

### Serum parameters and cognition by ApoE genotype in T2DM-MCI subjects

After grouping the T2DM-MCI subjects according to *ApoE* genotypes, we observed significant difference of cognitive function by *ApoE* genotypes. As shown in Table 7, comparing with *E2* and *E3* carriers, the *E4* subjects have lower cognitive abilities in visual & executive, memory and delayed recall and orientation domains (*P* < 0.05). Also, the global cognition (total MoCA score) of *ApoE4* carrier was lower than *E2* carriers and those with *E3* homozygote (*P* < 0.05). No difference of serum parameters by *ApoE* genotype was observed in T2DM-MCI subjects (*P* < 0.05).

### Combine effect of ApoE genotype and T2DM on serum parameters and cognition in MCI subjects

The combine effects of *ApoE* genotype and T2DM on serum parameters and cognition was detected in MCI subjects (Table 8). Among the MCI subjects, the lowest orientation ability and total MoCA score were observed in T2DM-MCI subjects carrying *ApoE4*; and the interaction of *ApoE* genotype with T2DM was detected (*P* < 0.05). No combine effect of *ApoE* genotype and T2DM on serum parameters in MCI subjects was observed (*P* > 0.05).

**Table 3:** Serum parameters, ApoE genotype and cognition in MCI subjects with or without T2DM.

Parameters, genotype and cognition	nT2DM-MCI *(n = 168)*	T2DM-MCI *(n = 43)*	*P* value
Serum Parameters (mmol/L)			
*Glu*	5.8 (5.5, 6.0)	7.5 (7.0, 7.9)	0.00
*TC*	5.3 (5.1, 5.5)	5.2 (4.8, 5.5)	0.61
*TG*	1.8 (1.6, 1.9)	2.0 (1.6, 2.3)	0.29
*LDL-C*	3.1 (3.0, 3.2)	3.2 (2.9, 3.5)	0.51
*HDL-C*	1.5 (1.4, 1.5)	1.3 (1.2, 1.4)	0.00
*ApoE* genotype, n (%)			0.81
*E2*	34 (20.2)	6 (14.0)	
*E3*	111 (66.1)	31 (72.1)	
*E4*	23 (13.7)	6 (14.0)	
Cognition			
*Visual & executive*	3.0 (2.8, 3.2)	2.6 (2.2, 3.0)	0.09
*Naming*	2.7 (2.6, 2.8)	2.6 (2.4, 2.9)	0.61
*Attention*	4.3 (4.0, 4.5)	4.6 4.1, 5.1)	0.27
*Language*	1.3 (1.2, 1.5)	1.6 (1.4, 1.9)	0.07
*Abstraction*	1.0 (0.8, 1.1)	1.0 (0.7, 1.2)	0.98
*Memory and delayed recall*	1.5 (1.3, 1.7)	1.5 (1.0, 1.9)	0.88
*Orientation*	5.3 (5.0, 5.5)	5.3 (4.9, 5.8)	0.78
*MoCA score*	19.2 (18.3, 20.1)	19.4 (17.5, 21.2)	0.86

Data were expressed as mean (95% *CI*). Glu, glucose; HDL-C, high density lipoprotein cholesterol; LDL-C, low density lipoprotein cholesterol; TC, total cholesterol; TG, triglyceride; ApoE, apolipoprotein E; T2DM: type 2 diabetes mellitus; MCI, mild cognitive impairment. *ApoE* genotype frequencies were compared by using Chi-square test. General Line Model (GLM) was used for data serum parameters and cognition comparison. During the comparison of serum parameters and cognition between groups, factors including age, BMI, education, smoking, alcohol drinking, exercise, reading habits, housework doing were adjusted. *P* < 0.05 was considered as significance.

**Table 4:** Dietary intake of MCI subjects with or without T2DM.

Foods (g/d)	nT2DM-MCI *(n = 168)*	T2DM-MCI *(n = 43)*	*P* value
Fruit	154.5 (139.2, 169.8)	121.4 (90.3, 152.6)	0.06
Vegetable	289.8 (270.1, 309.4)	277.2 (237.2, 317.2)	0.58
Fruit + Vegetable	444.3 (417.7, 470.8)	398.6 (344.6, 452.7)	0.14
legume	29.0 (25.2, 32.8)	18.65 (10.9, 26.4)	0.02
Cooking oil	30.6 (28.0, 33.2)	31.18 (25.9, 36.4)	0.85
Fish	19.4 (17.2, 21.6)	17.31 (12.8, 21.8)	0.41
Whole grain	27.7 (24.9, 30.4)	26.1 (20.5, 31.6)	0.61
Red meat	27.9 (24.0, 31.8)	21.5 (13.5, 29.5)	0.16
Poultry	13.3 (11.2, 15.4)	11.5 (7.2, 15.8)	0.47
Nut	12.7 (10.4, 15.0)	10.6 (5.9, 15.3)	0.43
Milk	191.5 (175.8, 207.3)	200.8 (168.6, 232.9)	0.62
Egg	30.2 (27.7, 32.7)	32.1 (27.0, 37.2)	0.52

Data were expressed as mean (95% *CI*). nT2DM-MCI: mild cognitive impairment subjects without type 2 diabetes mellitus; T2DM-MCI: mild cognitive impairment subjects with type 2 diabetes mellitus. General Line Model (GLM) was used for data analysis. Factors including sex, age, BMI and exercise were adjusted. *P* < 0.05 was considered as significance.

**Table 5:** Serum parameters and cognition according to *ApoE* genotype in T2DM patients.

Serum parameters and cognition	*ApoE E2 (n = 29)*	*ApoE3 (n = 176)*	*ApoE4 (n = 33)*	*P* value
Serum Parameters (mmol/L)				
*Glu*	7.2 (6.3, 8.1)	7.2 (6.8, 7.6)	7.3 (6.4, 8.2)	0.98
*TC*	5.0 (4.5, 5.4)	4.9 (4.7, 5.0)	4.9 (4.5, 5.3)	0.35
*TG*	2.5 (1.8, 3.1)	1.8 (1.6, 2.1)	2.6 (2.0, 3.2)	0.04
*LDL-C*	2.9 (2.5, 3.3)	3.2 (3.0, 3.3)	3.1 (2.7, 3.4)	0.40
*HDL-C*	1.3 (1.1, 1.4)	1.3 (1.3, 1.4)	1.2 (1.1, 1.3)	0.44
Cognition				
*Visual & executive*	3.9 (3.2, 4.5)	3.7 (3.2, 4.1)	3.3 (2.9, 3.8)	0.10
*Naming*	2.9 (2.7, 3.2)	2.9 (2.9, 3.0)	2.8 (2.6, 2.9)	0.14
*Attention*	5.5 (5.1, 5.9)	5.4 (5.2, 5.6)	5.2 (4.8, 5.6)	0.73
*Language*	2.4 (2.1, 2.7)	2.2 (2.1, 2.4)	1.9 (1.6, 2.2)	0.08
*Abstraction*	1.7 (1.5, 2.0)	1.6 (1.5, 1.8)	1.6 (1.4, 1.9)	0.01
*Memory and delayed recall*	2.7 (2.2, 3.3)	3.2 (2.9, 3.4)	2.9 (2.4, 3.5)	0.12
*Orientation*	5.8 (5.6, 6.1)	5.8 (5.7, 5.9)	5.54 (5.3, 5.8)	0.24
*MoCA score*	25.2 (23.6, 26.8)	25.5 (24.8, 26.1)	23.6 (22.1, 25.0)	0.04

Data were expressed as mean (95% *CI*). Glu, glucose; HDL-C, high density lipoprotein cholesterol; LDL-C, low density lipoprotein cholesterol; TC, total cholesterol; TG, triglyceride; ApoE, apolipoprotein E; General Line Model (GLM) was used for data serum parameters and cognition comparison. During the comparison of serum parameters and cognition between groups, factors including age, BMI, education, smoking, alcohol drinking, exercise, reading habits, housework doing were adjusted. *P* < 0.05 was considered as significance; and *P* value stand for the statistical significance between different *ApoE* genotype groups.

## DISCUSSION

Higher MCI prevalence was observed in T2DM patients than in the general population [28, 29]. Patients with both T2DM and MCI had a faster cognitive deterioration process than patients with MCI only [30]. All these data indicated that T2DM is a risk factor for the old population to have cognition decline. In the present study, we carried out a community based cross-sectional study trying to explore the association of ApoE genetic polymorphism, T2DM and cognition in non-demented aging Chinese adults. In the present study, we detected the *ApoE* genotype difference of cognitive performance in subjects with T2DM. The *ApoE4*-T2DM subjects have the lowest cognition as comparing with T2DM subjects carrying *E2* or *E3*. Moreover, the carrying of *ApoE4* predisposed the T2DM-MCI subjects to have much poor cognitive performance.

Cognition and serum parameter levels were compared between the T2DM and nT2DM subjects. Our data did not implicate obvious difference of cognitive function between theT2DM and nT2DM subjects (*P* > 0.05) (Table 1 & 2). This result was not in agreement with some previous studies [31,32]. Except of differences in study design and the method used for assessing cognitive function, the discrepancies between our and others’ studies might result from the inclusion of participants (all participants in the present study were community dwellers without clinically diagnosable cognition decline or dementia).

It is well known that glucose metabolism disorder (hyperglycemia) was the typical diabetic phenotype of T2DM patients. Hyperglycemia was suggested one of the mechanisms contributing to the cognition decline in diabetes. Chronic exposure to high levels of glucose could cause selective death of neurons, which terminally leads to cognitive deficits [33]. Abnormal lipid profile in T2DM patients was also implicated by previous studies [34,35]. Windler and Taskinen also reported that, compared to nT2DM individuals, markedly higher triglycerides and moderately lower HDL levels were detected in the diabetes patients [36,37]. In the current study, notable difference of serum Glu, TG and HDL-C levels were observed among participants with and without diabetes (*P* < 0.05). The largest disparity in serum glucose and lipid levels observed in T2DM and nT2DM subjects indicating the impaired of glucose and lipids metabolism in T2DM subjects. The serum parameter levels and cognition in MCI subjects with or without T2DM were also compared (Table 3). Although, no statistical significant difference of cognition by T2DM was observed, we founded that the serum lipid profile was obviously difference between the T2DM-MCI and nT2DM-MCI subjects. The T2DM-MCI subjects had higher serum Glu level and lower HDL-C level than nT2DM-MCI subjects (*P* < 0.05). According to previous study, a higher prevalence of T2DM or impaired glucose metabolism in patients with AD than in control subjects [38], which supports the involvement of impaired glucose metabolism in AD development. Regarding serum lipid profile, it was efficiency influenced by dietary fat-containing foods intakes. Thus, the comparison of dietary intakes of fat-containing foods between T2DM-MCI and nT2DM-MCI subjects might help us to uncover the underlying mechanism contributing to the observed inconsistency of serum lipid profile. No significant difference of dietary fat-containing foods intake was founded between T2DM-MCI and nT2DM-MCI subjects (*P* > 0.05). Our data only implicated the relative lower legume (a protein rich food) intake in T2DM-MCI subjects than nT2DM MCI subjects (Table 4). This result indicated that, except of diet, some other factors might contribute to the significant different lipid profile between T2DM-MCI and nT2DM-MCI subjects.

**Table 6:** Combine effect of ApoE genotype and T2DM on serum parameters and cognition in aging Chinese subjects.

Serum parameters and cognition	*ApoE2* (*n=134*)		*ApoE3* (*n=673*)		*ApoE4* (n=145)		*P* value

	nT2DM (*n=105*)	T2DM (*n=29*)	nT2DM (*n=497*)	T2DM (*n=176*)	nT2DM (*n=112*)	T2DM (*n=33*)	
Serum parameter (mmol/L)							
*Glu*	5.5 (5.2, 5.9)	7.3 (6.7, 7.9)	5.4 (5.2, 5.5)	7.2 (7.0, 7.5)	5.2 (4.9, 5.6)	7.3 (6.7, 8.0)	0.30
*TC*	5.2 (4.9, 5.4)	5.0 (4.6, 5.4)	5.0 (4.9, 5.1)	4.9 (4.7, 5.1)	5.2 (5.0, 5.4)	5.0 (4.6, 5.4)	0.97
*TG*	2.2 (1.9, 2.5)	2.6 (2.0, 3.07)	1.7 (1.5, 1.8)	1.9 (1.6, 2.1)	1.8 (1.5, 2.0)	2.6 (2.1, 3.1)	0.21
*LDL-C*	2.9 (2.7, 3.1)	2.9 (2.6, 3.3)	3.2 (3.1, 3.3)	3.2 (3.1, 3.3)	3.3 (3.1, 3.5)	3.1 (2.8, 3.4)	0.55
*HDL-C*	1.5 (1.4, 1.6)	1.3 (1.1, 1.4)	1.4 (1.3, 1.4)	1.3 (1.3, 1.4)	1.4 (1.4, 1.5)	1.2 (1.1, 1.3)	0.05
Cognition							
*Visual & executive*	3.8 (3.6, 4.1)	3.6 (3.2, 4.1)	3.9 (3.8, 4.0)	4.0 (3.8, 4.1)	4.0 (3.7, 4.2)	3.4 (3.0, 3.8)	0.14
*Naming*	2.9 (2.8, 3.0)	2.9 (2.7, 3.0)	2.9 (2.9, 3.0)	2.9 (2.9, 3.0)	2.8 (2.8, 2.9)	2.8 (2.6, 2.9)	0.63
*Attention*	5.2 (4.9, 5.4)	5.5 (5.1, 6.0)	5.3 (5.2, 5.4)	5.5 (5.3, 5.6)	5.4 (5.2, 5.6)	5.2 (4.8, 5.6)	0.46
*Language*	2.1 (1.9, 2.3)	2.4 (2.1, 2.7)	2.2 (2.2, 2.3)	2.3 (2.1, 2.4)	2.2 (2.0, 2.3)	2.0 (1.7, 2.3)	0.24
*Abstraction*	1.6 (1.4, 1.7)	1.8 (1.5, 2.0)	1.7 (1.6, 1.7)	1.7 (1.5, 1.8)	1.6 (1.5, 1.7)	1.6 (1.4, 1.8)	0.64
*Memory and delayed recall*	3.1 (2.8, 3.4)	2.7 (2.2, 3.3)	3.1 (2.9, 3.2)	3.2 (3.0, 3.4)	3.0 (2.7, 3.3)	3.0 (2.5, 3.5)	0.42
*orientation*	5.8 (5.6, 5.9)	5.9 (5.6, 6.18)	5.8 (5.7, 5.9)	5.9 (5.7, 6.0)	5.7 (5.6, 5.9)	5.5 (5.2, 5.8)	0.48
*MoCA score*	24.5 (23.6, 25.4)	25.3 (23.5, 27.0)	25.1 (24.7, 25.5)	25.7 (25.0, 26.4)	24.9 (24.0, 25.8)	23.7 (22.1, 25.3)	0.38

Data were expressed as mean (95% *CI*). Glu, glucose; HDL-C, high density lipoprotein cholesterol; LDL-C, low density lipoprotein cholesterol; TC, total cholesterol; TG, triglyceride; ApoE, apolipoprotein E; nT2DM-MCI, mild cognitive impairment subjects without type 2 diabetes mellitus; T2DM-MCI, mild cognitive impairment subjects with type 2 diabetes mellitus. General Line Model (GLM) was used for data serum parameters and cognition comparison. Factors including age, BMI, education, smoking, alcohol drinking, exercise, reading habits, housework doing were adjusted. *P* < 0.05 was considered as significance.

**Table 7:** Serum parameters and cognition according to *ApoE* genotype in T2DM-MCI subjects.

Serum parameters and cognition	*ApoE2 (n = 6)*	*ApoE3 (n = 31)*	*ApoE4 (n = 6)*	*P* value
Serum Parameters (mmol/L)
*Glu*	6.7 (4.8, 8.6)	7.5 (6.7, 8.3)	8.9 (6.9, 11.0)	0.43
*TC*	4.7 (3.5, 5.9)	5.0 (4.5, 5.5)	5.5 (4.2, 6.8)	0.53
*TG*	1.6 (0.2, 3.0)	2.2 (1.6, 2.8)	1.9 (0.4, 3.4)	0.57
*LDL-C*	2.4 (1.3, 3.5)	3.1 (2.7, 3.6)	3.2 (2.0, 4.3)	0.36
*HDL-C*	1.4 (1.2, 1.7)	1.2 (1.1, 1.3)	1.1 (0.9, 1.4)	0.25
Cognition				
*Visual & executive*	2.6 (1.4, 3.8)	2.6 (2.1, 3.1)	0.6 (-0.6, 1.9)	0.01
*Naming*	2.8 (2.1, 3.5)	2.8 (2.5, 3.1)	1.6 (0.9, 2.4)	0.06
*Attention*	4.2 (2.7, 5.8)	4.6 (3.9, 5.2)	3.2 (1.6, 4.8)	0.42
*Language*	2.2 (1.3, 3.1)	1.7 (1.3, 2.0)	0.5 (-0.5, 1.4)	0.09
*Abstraction*	0.8 (0.0, 1.6)	1.2 (0.9, 1.5)	0.2 (-0.6, 1.1)	0.07
*Memory and delayed recall*	0.5 (-0.1, 1.9)	1.9 (1.3, 2.4)	0.2 (-1.1, 1.6)	0.04
*Orientation*	5.3 (4.2, 6.4)	5.6 (5.1, 6.0)	3.6 (2.4, 4.7)	0.02
*MoCA score*	18.1 (13.2, 23.0)	20.5 (18.5, 22.5)	10.5 (5.3, 15.6)	0.01

Data were expressed as mean (95% *CI*). ApoE: apolipoprotein E. General Line Model (GLM) was used for data serum parameters and cognition comparison. During the comparison of serum parameters and cognition between groups, factors including age, BMI, education, smoking, alcohol drinking, exercise, reading habits, housework doing were adjusted. *P* < 0.05 was considered as significance; and *P* value stand for the statistical significance between different *ApoE* genotype groups.

*ApoE ε4* is an established risk factor for late-onset Alzheimer’s disease. Moreover, the development of T2DM was suggested to be associated with *ApoE* genotype [39]. Therefore, in the current study, we try to explore whether there was synergistic effect of *ApoE* genotype and T2DM on cognition in non-demented aging adults. Although the combine effect of *ApoE* genotype with T2DM in affecting cognitive function and serum parameters was not observed, we founded that serum TG level and several cognitive domains (including abstraction domain and global cognition) in T2DM subjects were associated with *ApoE* genotype. As comparing with nT2DM subjects, higher serum TG level was detected in T2DM subjects carrying *ApoE4* or *ApoE2*, which implicating the susceptibility of serum TG concentration to genetic variant of *ApoE* in T2DM patients. Moreover, for *ApoE4*-T2DM subjects, the effect of ApoE on serum TG status became more remarkable (Table 5). Our results are consistent with the study carried out in Thai population. In which, the researcher founded that *ApoE ε4* allele-diabetic carriers showed a significantly higher serum TG and lower HDL-C levels compared to *E3/E3* genotype carriers [40]. Additionally, *ApoE ε4* allele was also reported to be associated with higher serum LDL-C and lower HDL-C levels in Spanish with T2DM [41]. Totally, these results suggested that, for T2DM subjects, the carrying of *ApoE ε4* allele implicate a predisposition of lipid metabolism disorder and cognition decline.

**Table 8:** Combine effect of *ApoE* genotype and T2DM on serum parameters and cognition in MCI subjects,

Parameters and cognition	*ApoE2* (*n=40*)		*ApoE3* (*n=142*)		*ApoE4* (*n=29*)		*P* value

	nT2DM-MCI (*n=34*)	T2DM-MCI (*n=6*)	nT2DM-MCI (*n=111*)	T2DM-MCI (*n=31*)	nT2DM-MCI (*n=23*)	T2DM-MCI (*n=6*)	
Serum parameters (mmol/L)							
*Glu*	5.8 (5.2, 6.4)	6.7 (4.8, 8.6)	5.6 (5.3, 5.9)	7.5 (6.7, 8.3)	5.8 (5.1, 6.5)	8.9 (6.9, 11.0)	0.37
*TC*	5.5 (5.1, 5.9)	4.7 (3.5, 5.9)	5.1 (4.9, 5.4)	5.0 (4.5, 5.5)	5.9 (5.3, 6.4)	5.5 (4.2, 6.8)	0.29
*TG*	2.0 (1.6, 2.5)	1.6 (0.2, 3.0)	1.6 (1.4, 1.8)	2.2 (1.6, 2.8)	1.9 (1.4, 2.5)	1.9 (0.4, 3.4)	0.14
*LDL-C*	2.9 (2.6, 3.3)	2.4 (1.3, 3.5)	3.1 (2.9, 3.3)	3.1 (2.7, 3.6)	3.4 (3.0, 3.9)	3.2 (2.0, 4.3)	0.14
*HDL-C*	1.6 (1.5, 1.7)	1.4 (1.2, 1.7)	1.6 (1.5, 1.7)	1.2 (1.1, 1.3)	1.4 (1.4, 1.5)	1.1 (0.9, 1.4)	0.87
Cognition							
*Visual & executive*	3.3 (2.8,3.8)	2.6 (1.4, 3.8)	3.0 (2.7, 3.3)	2.6 (2.1, 3.1)	2.7 (2.1, 3.3)	0.6 (-0.6, 1.9)	0.09
*Naming*	2.8 (2.5, 3.0)	2.8 (2.1, 3.5)	2.8 (2.6, 2.9)	2.8 (2.5, 3.1)	2.3 (1.9, 2.6)	1.6 (0.9, 2.4)	0.18
*Attention*	4.4 (3.8, 5.0)	4.3 (2.7, 5.8)	4.2 (3.8, 4.5)	4.6 (3.9, 5.2)	3.9 (3.2, 4.6)	3.2 (1.6, 4.8)	0.39
*Language*	1.3 (1.1, 1.7)	2.2 (1.3, 3.1)	1.3 (1.1, 1.5)	1.7 (1.3, 2.0)	1.1 (0.8, 1.5)	0.5 (-0.5, 1.4)	0.06
*Abstraction*	0.8 (0.5, 1.1)	0.8 (0.0, 1.6)	1.1 (0.9, 1.3)	1.2 (0.9, 1.5)	0.9 (0.5, 1.2)	0.2 (-0.6, 1.1)	0.32
*Memory and delayed recall*	1.6 (1.2, 2.1)	0.5 (-1.0, 1.9)	1.6 (1.3, 1.8)	1.7 (1.3, 2.4)	1.6 (1.0, 2.2)	0.2 (-1.1, 1.6)	0.20
*orientation*	5.2 (4.7, 5.8)	5.3 (4.2, 6.4)	5.2 (4.9, 5.5)	5.6 (5.1, 6.0)	5.0 (4.4, 5.6)	3.6 (2.4, 4.7)*	0.01
*MoCA score*	19.6 (17.5, 21.7)	18.1 (13.2, 23.0)	19.3 (18.1, 20.5)	20.5 (18.5, 22.5)	17.6 (15.0, 20.1)	10.5 (5.3, 15.1)*	0.03

Data were expressed as mean (95% *CI*). ApoE: apolipoprotein E; nT2DM-MCI, mild cognitive impairment subjects without type 2 diabetes mellitus; T2DM-MCI, mild cognitive impairment subjects with type 2 diabetes mellitus. General Line Model (GLM) was used for data serum parameters and cognition comparison. During the comparison of serum parameters and cognition between groups, factors including age, BMI, education, smoking, alcohol drinking, exercise, reading habits, housework doing were adjusted. *P* < 0.05 was considered as significance; *P* value stands for the statistical significance of *ApoE* genotype and T2DM interaction.

* *P* < 0.05, comparing with other nT2DM or T2DM MCI subjects with different *ApoE* genotypes.

After grouping the T2DM-MCI subjects by *ApoE* genotype, we observed notable difference of cognition between groups. Previous researches have indicated that diabetes is related to decrements in several cognitive domains, including processing speed, executive functions and memory [42-44]. In the present study, the cognition of *ApoE4* carriers were characterized as much lower abilities in visual & executive, memory and delayed recall and orientation domains accompanying with decreased global cognition (total MoCA score) than subjects with other *ApoE* genotypes (Table 7). Additionally, the interaction of *ApoE* genotype and T2DM on cognition (especially on the orientation domain and global cognition) was detected (Table 8). Our findings suggest that T2DM might involve in cognitive change in an *ApoE* genotype-dependent way. The carrying of *ApoE ε4* allele might expose the T2DM subjects to the high risk of cognition decline. These findings were partially in line with previous studies. Increasing evidences indicated that T2DM have been associated with accelerated cognitive decline, and dementia among older adults [45]. Additionally, it was reported that T2DM and *ApoE ε4* allele synergistically increased the pathological changes, such as neuritic plaques in hippocampus, NFTs in the cortex and hippocampus, and amyloid angiopathy in the brain [46]. These results indicated that ApoE gene polymorphism might be associated with a wide range of pathophysiological changes in brain and terminally affecting the cognitive functional status in T2DM patients.

Some limitations of the present study should also be addressed. The relative small sample size is a major drawback of the current study, so the extrapolation of our results to others should be with caution. Moreover, the current study was a community-based cross-sectional study, lacking the capacity of causal inference. Although some covariates were adjusted during the data analysis, some residual confounding is possible. Without measuring T2DM and cognition related biomarkers, we are incapable to exploring the interactive mechanism underlying *ApoE* genotype, T2DM and cognition. Although these shortcomings, our results, combined with those from other studies, suggested that older individuals with T2DM and *ApoE* ε4 allele are at an increased risk of cognition decline. Additional experimental studies are required to test the hypothesis that *ApoE* genotype modifies the risk for cognitive impairment in aging subjects with T2DM.

## Conclusion

In summary, our data implicated the association among ApoE gene polymorphism, T2DM and cognition in non-demented aging Chinese adults. The *ApoE4*-T2DM subjects and *ApoE4*-T2DM-MCI subjects were predisposed to have poor cognitive performance. In the future, long time cohort studies were needed with regard to how genetic background modulates the association between diabetes and cognitive function in aging population. The precise biological mechanism underlying this significant association should be investigated.
